# Cellular Metabolic Profiling of CrFK Cells Infected with Feline Infectious Peritonitis Virus Using Phenotype Microarrays

**DOI:** 10.3390/pathogens9050412

**Published:** 2020-05-25

**Authors:** Shing Wei Ng, Gayathri Thevi Selvarajah, Yoke Kqueen Cheah, Farina Mustaffa Kamal, Abdul Rahman Omar

**Affiliations:** 1 Department of Veterinary Clinical Studies, Faculty of Veterinary Medicine, Universiti Putra Malaysia, UPM Serdang, Selangor 43400, Malaysia; shingwei89@gmail.com; 2 Department of Biomedical Sciences, Faculty of Medicine and Biomedical Health Sciences, Universiti Putra Malaysia, UPM Serdang, Selangor 43400, Malaysia; ykcheah@upm.edu.my; 3 Department of Veterinary Pathology and Microbiology, Faculty of Veterinary Medicine, Universiti Putra Malaysia, UPM Serdang, Selangor 43400, Malaysia; farina@upm.edu.my (F.M.K.); aro@upm.edu.my (A.R.O.); 4 Institute of Bioscience, Universiti Putra Malaysia, UPM Serdang, Selangor 43400, Malaysia

**Keywords:** feline infectious peritonitis virus, cellular metabolism, phenotype microarray, metabolic profiling, glutamine, CrFK cells

## Abstract

Feline infectious peritonitis (FIP) is a fatal feline immune-mediated disease caused by feline infectious peritonitis virus (FIPV). Little is known about the biological pathways associated in FIP pathogenesis. This is the first study aiming to determine the phenotypic characteristics on the cellular level in relation to specific metabolic pathways of importance to FIP pathogenesis. Methods: The internalization of type II FIPV WSU 79-1146 in Crandell-Rees Feline Kidney (CrFK) cells was visualized using a fluorescence microscope, and optimization prior to phenotype microarray (PM) study was performed. Then, four types of Biolog Phenotype MicroArray™ plates (PM-M1 to PM-M4) precoated with different carbon and nitrogen sources were used to determine the metabolic profiles in FIPV-infected cells. Results: The utilization of palatinose was significantly low in FIPV-infected cells; however, there were significant increases in utilizing melibionic acid, L-glutamine, L-glutamic acid and alanyl-glutamine (Ala-Gln) compared to non-infected cells. Conclusion: This study has provided the first insights into the metabolic profiling of a feline coronavirus infection in vitro using PMs and deduced that glutamine metabolism is one of the essential metabolic pathways for FIPV infection and replication. Further studies are necessary to develop strategies to target the glutamine metabolic pathway in FIPV infection.

## 1. Introduction

The recent worldwide outbreak of a novel coronavirus, namely coronavirus disease 2019 (COVID-19), and the sudden emergence of coronavirus-related diseases in past decades has greatly attracted the attention of researchers in uncovering the pathophysiology and pathogenesis of coronaviruses to expedite the progress in finding effective antiviral treatments [1,2]. Coronaviruses are enveloped, single-stranded, positive-sense RNA viruses and differentiated into four groups based on their genetic and serological properties, and these viruses are known to cause acute and chronic respiratory disease, central nervous system disease and gastroenteritis in animals and human [2]. Feline coronavirus (FCoV) is an omnipresent virus that infects cats and is primarily transmitted through the fecal–oral route. FCoV is characterized as an Alpha-coronavirus—a category in which transmissible gastroenteritis virus and human coronavirus 229E (HCoV-229E) are also grouped [3].

FCoV consists of two different biotypes: feline enteric coronavirus (FECV) and feline infectious peritonitis virus (FIPV). Cats infected with FECV usually are asymptomatic or sometimes with mild enteritis (self-limiting diarrhoea), while FIPV causes a lethal immune-mediated feline disease which is feline infectious peritonitis (FIP) [4]. FCoV also consists of two serotypes: type I FCoV and type II FCoV. Type I FCoV is the dominant strain in the field. In contrast, type II FCoV is associated with canine coronavirus genetically, and it is studied mostly in vitro due to its greater propensity to proliferate in cell culture [3].

According to the “internal mutation theory”, FIPV arises when the mutation occurs in FECV mainly associated with the accessory and structural genes, namely ORF 3c accessory gene and spike gene, which are correlated with the virus virulence [5,6,7]. A high level of FCoV infection rate among the domesticated cats, especially in multi-cat households and shelters, are likely to develop FIP as cats who have recovered from the FCoV infection are generally susceptible to re-infection with the same or different strains of FCoV [3,5]. The most common form of FIP is effusive (wet, non-parenchymatous), where the inflammation observed in viscera, serosa and omentum with ascites [4]. Cats with pyogranulomatous or granulomatous lesions in multiple organs, including kidneys, liver and central nervous system, are characterized as non-effusive (dry, parenchymatous) FIP [4,8]. Cats with dry-form FIP have the possibility to develop into wet-form FIP as a result of their collapsed immune system. Furthermore, the majority of infected cats eventually succumb to FIP, primarily due to excessive host inflammatory response [5].

Flaviviruses and herpesviruses are among the most-discussed viruses in remodelling the host cellular metabolism, particularly in glycolysis and lipid biosynthesis to create an optimal viral replication and survival environment [9,10,11]. Dengue virus requires glucose and induces glycolysis for optimal viral replication; where studies have shown that infected cells have increased cellular glucose concentration over time compared to non-infected cells [9]. Latent infection of Kaposi’s Sarcoma-associated herpesvirus in endothelial cells activates the Warburg effect, changes in the glycolysis activity found in most cancer cells [10]. Fatty acid synthesis and glutaminolysis are also induced in the infected endothelial cells to ensure the rapid production of viral particles [10]. Glycoproteins and fatty acids are the examples of crucial macromolecules involved in the process of envelopment and the assembly of viral particles; the virus-induced cellular metabolism promotes the biosynthesis of these macromolecules for the benefit of these viruses [12]. Furthermore, modifications to cellular metabolism may prolong the survivability of the infected cells upon stress [12].

Apart from creating desirable conditions for viral growth, viral alteration on the central metabolic states of the infected cells regulates the biosynthesis of metabolic components, which are essential for innate immune response, this phenomenon might indirectly affect the inflammatory cytokine production of macrophages and other immune cells [13]. Metabolic reprogramming in glycolysis was found to be associated with the activation of macrophages and dendritic cells. Lipid biosynthesis modulates the production of immune mediators and this might affect the activity of macrophages as they need fatty acids for cytokine production [13,14,15]. Although evidence on coronavirus-induced metabolism is scarce, a study has reported that the rapid activation of the p38 mitogen-activated protein kinases (MAPK) pathway was detected in FIPV-infected primary blood-derived feline mononuclear cells, and this activation greatly regulates the production of pro-inflammatory cytokine (tumor necrosis factor (TNF)-alpha and interleukin (IL)-1 beta) during infection [16]. Further studies could be necessary to investigate the relationship between alterations of the host metabolism and the mechanism involved in immune responses during coronavirus infection.

The phenotype microarray (PM) represents the third major technology; in “phenomics”, alongside DNA microarrays and proteomic technologies, which is essential in this genomic era. A broad spectrum of applications is provided by PMs, such as determining gene functions and comparing gene knock-out mutants to wild types in microbial cells, identifying novel antimicrobial targets by finding genes unique to pathogenic microorganisms, and general cell characterization [17]. Most of the research using PMs has been focused on the analysis of metabolic phenotypes in cancer cells and bacterial infections, and it has rarely been used for viral infections [18,19]. In this study, the Biolog Phenotype MicroArray™ for mammalian cells (PM-M1 to PM-M4) consisting of 367 pre-existing metabolites was used to evaluate the utilization of various carbon and nitrogen sources in FIPV-infected cells, thus predicting the metabolic pathways involved in an FIPV infection.

## 2. Results

### 2.1. Internalization of FIPV WSU 79-1146 

**Figure 1:**
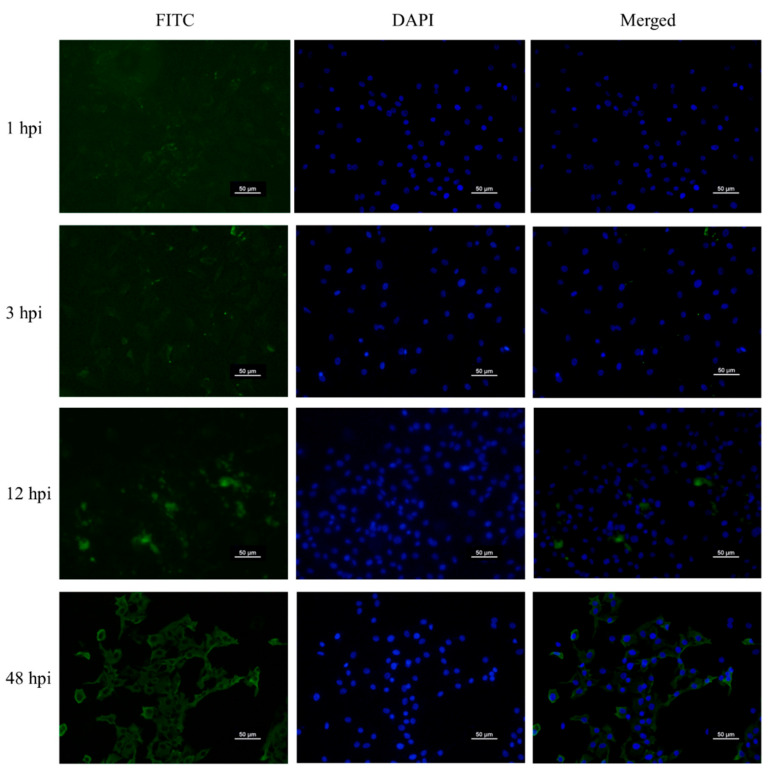
Representative fluorescence and merged microscopic images of internalization of feline infectious peritonitis virus (FIPV) WSU 79-1146 in Crandell-Rees Feline Kidney (CrFK) cells at different time intervals, up to 48 h. The nucleocapsid of FIPV was stained by fluorescein isothiocynate (FITC) and the nucleus of CrFK cells was stained by 4′,6-diamidino-2-phenylindole (DAPI). All images are in 200x magnification.

The internalization of FIPV WSU 79-1146 at a multiplicity of infection (MOI) of 1.0 in Crandell-Rees Feline Kidney (CrFK) cells at different hours post-infection (hpi) was observed with a fluorescence microscope (Figure 1). At 1 hpi, fluorescein isothiocynate (FITC)-conjugated antibody targeting the nucleocapsid of FIPV was detected at a very low intensity, which indicated that the entry of the virus into the cells was at the early stage. The intensity of FITC was gradually increased and observable in infected cells at 3 hpi. This might have indicated that the virus was moderately internalized into the cells after 3 hpi. The virus was internalized and replicated in the cells, which was represented by the high intensity of fluorescence observed at 48 hpi.

### 2.2. Detection of Intracellular Viral Load by SYBR Green-Based Real-Time PCR

The infection of FIPV in CrFK cells was detected based on the 3′UTR region of the FIPV genome using SYBR green-based real-time PCR. The intracellular viral load of infected cells was determined at different time points (Table 1). An increase in viral load along the hpi was noted and the highest viral load of 10^12.285^ was determined at 24 hpi in this study. The increasing trend of intracellular viral load indicates FIPV was replicated in the CrFK cells throughout the 24 hpi.

### 2.3. Effect of FBS Percentage on the Performance of Tetrazolium Dye

**Figure 2:**
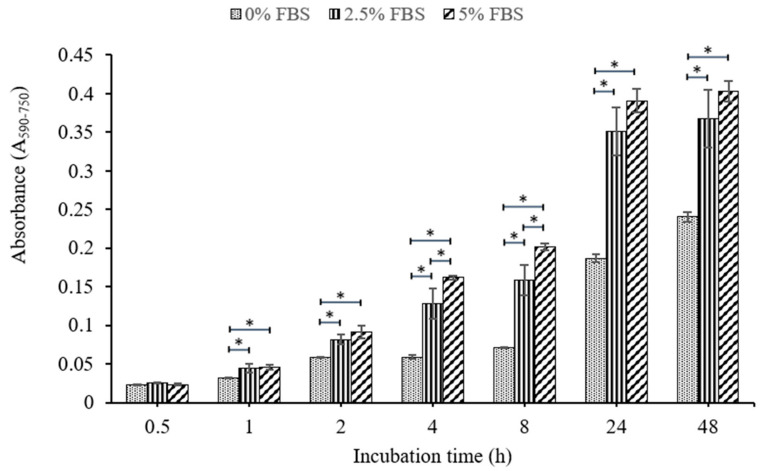
Absorbance (A_590–750_) comparison between different percentages of fetal bovine serum (FBS) (0%, 2.5% and 5%) in the incubation of CrFK cells at different time points, up to 48 h. The data represent the mean ± SD of three independent experiments. For each incubation period, means with * were significantly different (*p* < 0.05), from other FBS concentrations. Statistical analysis was performed by ANOVA test, followed by post hoc Tukey test.

Experiment to optimize the percentage of fetal bovine serum (FBS) use in the PM-M assays was performed, and the performance of tetrazolium dye in the cells at different incubation time is shown in Figure 2. When compared to 0% FBS, CrFK cells incubated with 2.5% and 5% FBS showed a significant increase in absorbance at 1 h. This also indicated that the cells could reduce tetrazolium as soon as 1 h after the incubation of the dye, up to 48 h. The absorbance difference was significant between 2.5% and 5% FBS used at 4 and 8 h, and the difference reflected that the metabolic activities of cells could be affected by the FBS percentages used in the assays. However, at total incubation periods of 24 and 48 h, there were no significant differences in absorbance among 2.5% and 5% FBS samples. Therefore, 2.5% of FBS was used in the media to culture CrFK cells for the subsequent PM-M assays.

### 2.4. Utilization of Carbon and Nitrogen Sources by the FIPV WSU 79-1146 Infected CrFK Cells

**Figure 3:**
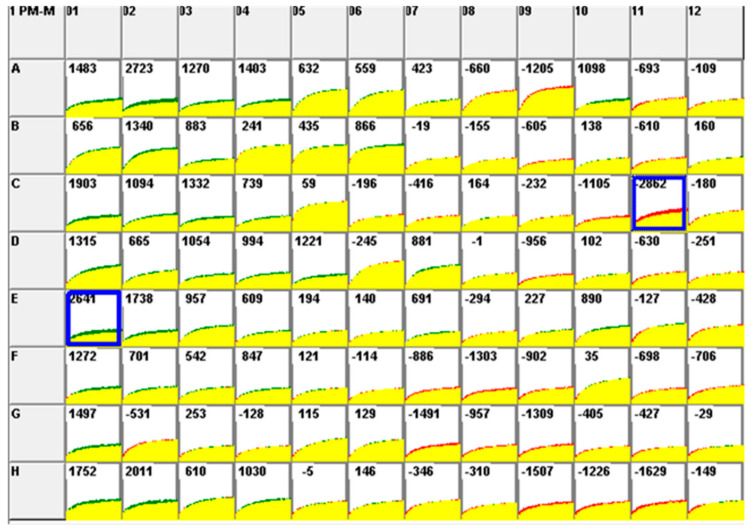
Comparison of metabolism rate between FIPV-infected CrFK cells (Green) and non-infected CrFK cells (Red) in PM-M1 plates for 24 hour post-infection (hpi). The yellow color indicates overlapping responses. The value displayed in each well indicates the difference of metabolism rate among the two assay conditions. The median value of this PM-M1 plate is 2343. Wells highlighted in Blue indicate significant metabolic activity in the corresponding metabolites among the two assay conditions (C11 = palatinose; E1 = melibionic acid).

According to the PM-M1 plate coated with carbohydrate and carboxylate substrates (Figure 3), virus-infected cells significantly inhibited the metabolism of palatinose, a disaccharide carbohydrate, for 24 hpi. However, significantly increased usage of melibionic acid was shown in infected cells compared to non-infected cells. 

**Figure 4:**
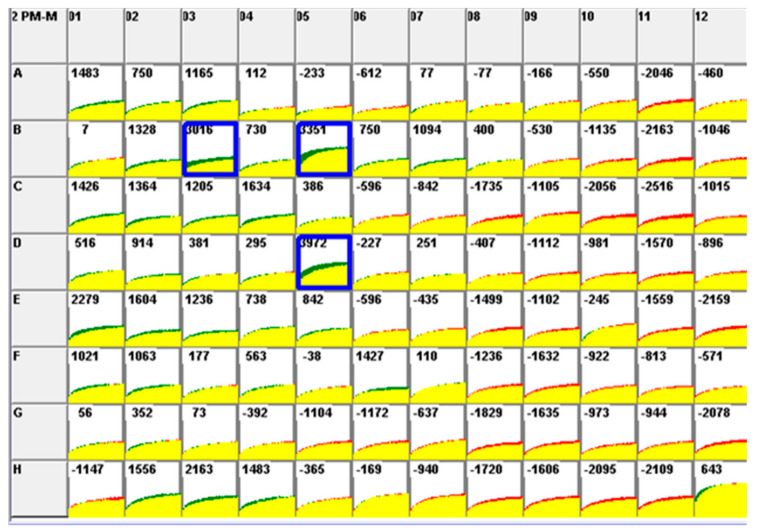
Comparison of metabolism rate between FIPV-infected CrFK cells (Green) and non-infected CrFK cells (Red) in PM-M2 plates for 24 hpi. The yellow color indicates overlapping responses. The value displayed in each well indicates the difference of metabolism rate among the two assay conditions. The median value of this PM-M2 plate is 2552. Wells highlighted in Blue indicate significant metabolic activity in the corresponding metabolites among the two assay conditions [B3 = L-glutamic acid; B5 = L-glutamine; D5 = alanyl-glutamine (Ala-Gln)].

The PM-M2 plate reflected a significant increase in utilizing two amino acids (L-glutamic acid, L-glutamine) and one dipeptide (alanyl-glutamine (Ala-Gln)) in virus-infected cells compared to non-infected cells for 24 hpi (Figure 4). PM-M3 and PM-M4 plates showed no significant metabolic activities in the tested metabolites between non-infected cells and infected cells.

The OmniLog^®^ (OL) PM software is designated to link with Kyoto Encyclopaedia of Genes and Genome (KEGG) databases. Many biological pathways involve L-glutamine and L-glutamic acid (Table 2); however, there is no depositary information related to palatinose, melibionic acid and Ala-Gln in KEGG databases.

## 3. Discussion

In this study, the optimum infection time of FIPV in CrFK cells for the PM experiment was determined at 3 hpi. A study on the kinetics of internalization for FIPV WSU 79-1146 has revealed that up to 70% of bound virus particles were internalized by CrFK cells at 3 hpi and longer incubation times increased the percentage of virus internalization via endocytosis [20]. FIPV-infected CrFK cells showed a sign of early apoptosis at 9 hpi followed by necrosis after 24 hpi [21]. An increase in intracellular virus load was detected at different hpi, this indicates that FIPV was replicated in the cells that correspond to the elevated FITC intensity observed in the cells. An in vitro replication kinetics of FCoV study has revealed the production of progeny virus in CrFK cells started from 3 hpi and increased exponentially up to 12 hpi, regardless of FCoV biotype [22]. Moreover, considering Redox Dye Mix MB, a tetrazolium dye used to perform PM-M assays required 5 to 24 h to develop color; 3 hpi was the suitable time to evaluate the metabolic profile of the FIPV-infected cells.

FBS is a necessity for the in vitro cell culture of eukaryotic cells. It contains essential growth factors for cell growth. Nevertheless, excessive usage of FBS in virus infection studies affects the binding affinity and infection efficiency of virus particles into cells, and the research suggests virus infection strategies have performed most efficiently when cultured without using FBS [23]. Several studies have also conceded that FBS inhibits influenza virus and hepatitis C virus attachment to host cells [24]. The data show a significant difference in absorbance at 4 and 8 h between 0%–5% of FBS used in medium, but not at 24 h. This indicates that the metabolic activities of CrFK cells that occurred between 4 and 8 h were significantly affected by the percentage of FBS despite there being no significant difference at 24 h. However, the virus binding inhibitory effect of FBS was taken into consideration, and 2.5% FBS was used in the subsequent PM-M assays.

The insights on the cellular mechanism involved in FIP pathogenesis have been discovered mainly via the transcriptional profiling of infected cell lines and peritoneal cells from cats experimentally infected with FIPV, but not typically on the cellular level [21,25,26]. This is the first study that aims to identify the metabolic profile of FIPV infection concerning certain biological pathways associated with FIP pathogenesis. The data indicated that the utilization of two disaccharide carbohydrates (palatinose and melibionic acid), two amino acids (L-glutamine and L-glutamic acid) and one dipeptide (Ala-Gln) was significantly affected in FIPV infection. Nonetheless, there is insufficiently detailed information with regards to cellular pathways involving palatinose, melibionic acid and Ala-Gln, either in KEGG databases or other related archives. Palatinose, also known as isomaltulose, is a naturally occurring disaccharide made up of α-1,6-linked glucose and fructose, and melibionic acid is a disaccharide derived from a melibiose, which consists of α-1,6-linked D-galactosyl and D-gluconic acid. Palatinose has been widely used as a non-cariogenic sucrose replacement and in products for diabetics and prediabetic dispositions [27]. In the current study, palatinose and melibionic acid could be alternative glucose sources for FIPV-induced cellular activities to produce energy. A similar explanation could be conceived for Ala-Gln, in that it might be a secondary source for the replacement of glutamine in cell metabolism. A study has shown that the supplementation of Ala-Gln to rats is able to act as an effective source of glutamine and regulate the muscle damage and inflammation due to prolonged exercise [28]. The healing process of muscle damage often disrupted by the chronic inflammatory response associated with the continual release of pro-inflammatory mediators (TNF-alpha and prostaglandin E2 (PGE2)) [28]. Ala-Gln supplementation has attenuated the plasma levels of TNF-alpha, PGE2 and creatine kinase (muscle damage biomarker) [28]. Another study has demonstrated that Ala-Gln could improve the pancreatic β-cell function via sirtuin 1/HUR signalling and further modulate the inflammatory mediators derived from macrophages [29]. Further clarifications on the usage of these substrates in an FIPV infection may require additional validation studies.

Glutamine and glucose are the two key carbon sources essential for mammalian cells’ growth. Glucose is converted to pyruvate and subsequently subjected to distinct pathways, including glycolysis and tricarboxylic acid (TCA) cycle, to produce energy. Similarly, glutamine involves multiple cellular metabolic pathways, such as arginine biosynthesis, nitrogen metabolism and, most importantly, glutaminolysis. Glutaminolysis is a process by which glutamine is metabolized into glutamate and finally to alpha-ketoglutarate—a key intermediate substrate for the TCA cycle [12]. It has been confirmed that viruses hijack the host cellular machinery and regulate the metabolic pathways for the biosynthesis of precursors components for efficient replication and production by utilizing glutamine [11]. Additionally, a study has pointed out that glutamine is greatly utilized in a vaccinia virus (VACV) infection compared to glucose, as glutamine is necessary for viral protein synthesis [30]. The authors detected that VACV protein synthesis was decreased in glutamine-deprived cells and deduced that the alteration of cellular metabolism by VACV is fully dependent on the presence of glutamine to maintain the TCA cycle [30]. The data show that FIPV utilized L-glutamine and L-glutamic acid (L-glutamate) in the absence of glucose for replication, and a significantly higher metabolic rate was observed in infected CrFK cells compared to non-infected CrFK cells. Despite promoting the viral replication, a study showed that the supplementation of glutamine could inhibit the reactivation of herpes simplex virus in mice and guinea pigs by upregulating the IFN-γ-associated immune response [31]. Additional research on the regulation of glutamine in virus infection could be considered to provide a deeper understanding of the importance of glutamine against coronaviruses.

Aside from the metabolic patterns of carbon and nitrogen substrates, the establishment of a lipidomic profile of a virus infection is also crucial. To determine the significance of lipid metabolism in relation to the pathogenesis of coronaviruses, a recent study has elucidated the lipid metabolism upon HCoV-229E infection by evaluating the levels of few lipid compounds including glycerophospholipids, fatty acids, linoleic acid (LA) and arachidonic acid (AA) in infected cells [32]. Interestingly, the metabolizing rate of glycerophospholipids and fatty acids increased while the utilization of LA and AA was significantly inhibited in HCoV-229E infection [32]. The authors also found that exogenous supplementation of LA and AA remarkably suppressed the replication of HCoV-299E and Middle East respiratory syndrome coronavirus [32].

This study highlights that glutamine metabolism plays an important role in a type II FIPV infection in CRFK cells, which could be a model for the in vivo situation. These findings also suggest that the metabolization of carbon and nitrogen sources are essential for coronavirus infection and pathogenesis. Further studies are required to evaluate the deprivation of glutamine metabolites in vitro and in vivo with type I FIPV infection as well, which are necessary as most of the acquired FIP clinical cases are mainly caused by the type I FIPV. Investigation on lipid metabolism in FIPV infection is necessary to outline strategies to target this virus during the infective or replication stages on the cellular level.

## 4. Materials and Methods

### 4.1. Reagents and Chemicals

All reagents and chemicals were purchased from Thermo Fisher Scientific (Waltham, MA, USA) unless otherwise noted.

### 4.2. Cell and Virus

CrFK cells and highly virulent type II FCoV strain FIPV WSU 79-1146 were purchased from American Type Culture Collection (ATCC) (Manassas, VA, USA). The virus was grown in confluent CrFK cells. The virus tissue culture infective dose 50 (TCID_50_) of FIPV WSU 79-1146 was calculated the using Reed–Muench method [33]. Briefly, a confluent 96-well plate of CrFK cells was inoculated with a serial 10-fold dilution of the virus and made up 100 µL for each virus dilution/well. The plate was incubated at 37 °C, 5% CO_2_. The formation of cytopathic effects was observed using an inverted microscope after 72 h of incubation, and the TCID_50_ was calculated.

### 4.3. Internalization of FIPV by Immunofluorescence

CrFK cells were seeded on eight-well chamber slides with a cell density of 4 × 10^4^/well and incubated for 24 h at 37 °C, 5% CO_2_. Then, the cells were infected with FIPV WSU 79-1146 at the MOI of 1.0 for predetermined times. At the definite infection time, a slide was removed from the incubator and processed. All the following procedures were performed at room temperature. The cells were rinsed with phosphate-buffered saline (PBS) and were fixed with neutral buffered 4% paraformaldehyde for 10 min. The cells were then rinsed with PBS three times with five min intervals. After that, 0.2% Triton X-100/PBS solution was added to permeabilize the cells for 10 min. The cells were rinsed again with PBS. The blocking buffer (5% goat serum in 1% FBS/PBS) was added into the cells and incubated for 30 min. Then, the blocking buffer was removed, and the cells were incubated with the mouse monoclonal anti-coronavirus antibody FIPV3-70 with 1:400 dilution for 30 min. Again, the cells were rinsed with PBS. Next, FITC-conjugated anti-mouse IgG2a secondary antibody diluted to 1:2000 with PBS was added to the cells and was incubated for 20 min in a dark environment. After that, the cells were rinsed three times with PBS. Lastly, Fluoroshield™ with DAPI (Sigma-Aldrich, St. Louis, MO, USA) mounting medium was added onto all the stained slides, covered with coverslip and sealed with nail polish. The slides were examined with a fluorescence microscope and digital camera system (Nikon Eclipse Ti-S, Nikon, Tokyo, Japan) and images were captured at different filters.

### 4.4. Detection of Intracellular Viral Load by SYBR Green-Based Real-Time PCR

CrFK cells were seeded on 24 -well plates with a cell density of 1 × 10^5^/well and incubated for 24 h at 37 °C, 5% CO_2_. Then, the cells were infected with FIPV WSU 79-1146 at the MOI of 1.0 for predetermined times. At the definite infection time, the cell pellets were collected via centrifugation at 700× *g* for 10 min (Allegra™ X22R Centrifuge, Beckman Coulter, Miami, FL, USA). Total RNA was extracted from the cell pellets using an RNeasy Mini Kit (Qiagen, Hilden, Germany) according to the manufacturer’s protocol. Then, the concentration and purity of extracted RNA were analyzed using Biospectrophotometer (Eppendorf, Hamburg, Germany). A total of 100 ng/µL RNA was used to synthesize cDNA using a Maxima First Strand cDNA Synthesis Kit, following the manufacturer’s protocol. Intracellular viral load was determined by SYBR green-based real-time PCR using primers targeting the 3′ untranslated region (UTR) of FIPV [34]. The quantitative real-time PCR (qPCR) was performed using Brilliant III Ultra-Fast SYBR Green Master Mix (Agilent, Santa Clara, CA, USA). Briefly, a volume of 20 µL reaction mix consists of 4 µL nuclease free water, 10 µL 2× brilliant III ultra-fast SYBR green qPCR master mix, 2 µL 5 µM forward primer, 2 µL 5 µM reverse primer and 10 ng of cDNA template. The qPCR reaction was performed on a CFX96 Touch™ Real-Time PCR Detection System (Bio-Rad, Hercules, CA, USA) with the following conditions: one cycle at 95 °C of initial denaturation for 2 min and 40 cycles of denaturation at 95 °C for 10 s, annealing at 60 °C for 15 s, and extension at 70 °C for 10 s. Detection of intracellular viral load was based on a standard curve generated from the serial dilution of a cDNA template synthesized form viral RNAs extracted from FIPV WSU 79-1146. 

### 4.5. Optimization of FBS Percentage on Performance on Tetrazolium Dye

Prior to determining the optimal culture condition to obtain acceptable background noises in PM-M assays, the optimization of the percentage of FBS used was necessary. To perform the assay, a cell suspension was prepared in an inoculating fluid deficient in carbon and energy source. In order to mimic the PM-M assay condition, an MC-0 assay medium containing 100 mL of Dulbecco’s Modified Eagle Medium (DMEM) without glucose, 1.1 mL of 100× Penicillin–Streptomycin solution, and 0.16 mL of 200 mM Glutamine (final concentration at 0.3 mM) was prepared and used for seeding cells. Briefly, 2 × 10^4^ cells/50 µL of CrFK cells were seeded into each well of a flat-bottom 96-well plate with different percentages of FBS in triplicate. The plates were incubated for 24 h in the humidifier incubator at 37 °C, 5% CO^2^ before adding 10 µL Redox Dye Mix MB (Biolog, Hayward, CA, USA) in the dark. The metabolic activity of cells could be examined via the reduction of the dye to a purple formazan which is captured by the Biolog Redox Dye color generation system or with a microplate reader. Color development was observed physically. The absorbance value of the color development at determined times in each well was quantified spectrophotometrically using a UV-Vis spectrophotometer (Tecan Infinite M200 PRO, Tecan, Austria) at 590 nm and 750 nm. The endpoint reads were obtained at 590 nm with a subtraction of a 750 nm reference reading (A_590–750_), which corrects for any background light scattering.

### 4.6. Phenotype Profiling of FIPV-Infected CrFK Cells Using PM-M Assays

The metabolic activity profiling of FIPV-infected CrFK cells was evaluated using PM-M (PM-M1 to PM-M4) plates where a total of 367 potential metabolic pathways were tested simultaneously. The list of metabolites for PM-M1, PM-M2, PM-M3 and PM-M4) are provided in Supplementary Materials (Figures S1–S4). The PM-M1 plate was coated with carbohydrate and carboxylate substrates while PM-M2, PM-M3 and PM-M4 plates were coated with differential L-amino acid and dipeptides. Briefly, CrFK cells were collected from a confluent 75 cm^2^ culture flask and resuspended in MC-0 assay medium. The cell number and cell viability were determined using a hemocytometer. Then, CrFK cells were seeded into the PM-M plates at 20,000 cells/well/50 µL, and the plates were incubated in a humidified incubator at 37 °C, 5% CO_2_ for 24 h. Next, 5 µL of FIPV inoculum at a MOI of 1.0 was added into each well of the PM-M plate, while an equal volume of medium without virus was added to another PM-M plate, which represented the non-infected control. The plates were incubated for 3 h at 37 °C, 5% CO_2_, before adding 10 µL Redox Dye Mix MB (Biolog, Hayward, CA, USA) in the dark. Next, the plates were quickly sealed with sterile plate sealer (Bio-rad, Hercules, CA, USA) and incubated in OL PM instrument (Biolog, Hayward, CA, USA) for 24 h. The reduction of tetrazolium was measured kinetically by the color CCD (charge-coupled device) digital camera of the OL PM instrument. Data were recorded in the PC connected to the instrument.

### 4.7. PM-M Assays Data Analysis

Data in D5E formats that were retrieved from the PC connected with OL incubator/reader were first converted to the OKA format by using file management and parametric suite software version 2009 (Biolog MicroLog™, Biolog, Hayward, CA, USA), then all the OKA format files were converted to the DLB format by using file management software (OL FM software, Biolog, Haward, CA, USA). The DLB format files were organized and separated into control and treated DLB format files. The average data value of the area under the kinetic curve from two independent experiments for each PM-M plate was generated by parametric software (OL PM software, Biolog, Hayward, CA, USA) and generated results between controls and treated samples were compared. A median (M) value of the area under the curve was calculated for each plate in order to determine significant wells of the PM-M plate. This median value was set in the OL PM software and significant wells with a higher value of the area under the curve than the calculated median was highlighted for each plate. The median value for each plate was calculated using the following equation:

(1) $$M=\frac{X+Y}{2}$$

where

M = median area under the curve for PM-M plate;

X = average area under the curve from negative control wells (A1 + A2 + A3);

Y = highest area under the curve (among sample wells).

### 4.8. Statistical Analysis

An analysis of variance (ANOVA) and *post hoc* Tukey test were performed using the SPSS version 20.0 software (Chicago, IL, USA) for the optimization test of the FBS percentage. A probability value of *p* < 0.05 was considered to indicate a statistically significant difference.

## Figures and Tables

**Table 1:** Intracellular FIPV titre in CrFK cells at different hpi.

Time Point (hpi)	Intracellular Virus Load (log10)
0	-
3	8.627 ± 0.003 ^a^
12	10.604 ± 0.012 ^b^
24	12.285 ± 0.048 ^c^

Data represent the mean ± SD of three independent experiments. ^a–c^ indicates significant different (*p* < 0.05). Statistical analysis was performed by ANOVA test, followed by post hoc Tukey test.

**Table 2:** Biological pathways involve both L-glutamine and L-glutamic acid.

Code in KEGG	Related-Pathways
map00220	Arginine biosynthesis
map00250	Alanine, aspartate and glutamate metabolism
map00471	D-Glutamine and D-glutamate metabolism
map00630	Glyoxylate and dicarboxylate metabolism
map00910	Nitrogen metabolism
map00970	Aminoacyl-tRNA biosynthesis
map01060	Biosynthesis of plant secondary metabolites
map01120	Microbial metabolism in diverse environments
map01230	Biosynthesis of amino acids
map02010	ABC transporters
map02020	Two-component system
map04724	Glutamatergic synapse
map04727	GABAergic synapse
map04964	Proximal tubule bicarbonate reclamation
map04974	Protein digestion and absorption
map05230	Central carbon metabolism in cancer

## Supplementary Materials

The following are available online at https://www.mdpi.com/2076-0817/9/5/412/s1, Figure S1: List of metabolites tested in PM-M1; Figure S2: List of metabolites tested in PM-M2; Figure S3: List of metabolites tested in PM-M3; Figure S4: List of metabolites tested in PM-M4.
