# Addendum: Ng, S.W. et al. Cellular Metabolic Profiling of CrFK Cells Infected with Feline Infectious Peritonitis Virus Using Phenotype Microarrays. *Pathogens* 2020, *9*, 412

**DOI:** 10.3390/pathogens9110931

**Published:** 2020-11-10

**Authors:** Shing Wei Ng, Gayathri Thevi Selvarajah, Yoke Kqueen Cheah, Farina Mustaffa Kamal, Abdul Rahman Omar

**Affiliations:** 1 Department of Veterinary Clinical Studies, Faculty of Veterinary Medicine, Universiti Putra Malaysia, UPM Serdang, Selangor 43400, Malaysia; shingwei89@gmail.com; 2 Department of Biomedical Sciences, Faculty of Medicine and Biomedical Health Sciences, Universiti Putra Malaysia, UPM Serdang, Selangor 43400, Malaysia; ykcheah@upm.edu.my; 3 Department of Veterinary Pathology and Microbiology, Faculty of Veterinary Medicine, Universiti Putra Malaysia, UPM Serdang, Selangor 43400, Malaysia; farina@upm.edu.my (F.M.K.); aro@upm.edu.my (A.R.O.); 4 Institute of Bioscience, Universiti Putra Malaysia, UPM Serdang, Selangor 43400, Malaysia

The authors wish to add another citation to the published paper [1]. The change is as follow:

Insert a citation to the paragraph in Section 4.7. PM-M Assays Data Analysis.

Data in D5E formats that were retrieved from the PC connected with the OL incubator/reader were first converted to the OKA format by using file management and parametric suite software version 2009 (Biolog MicroLog™, Biolog, Hayward, CA, USA), then all the OKA format files were converted to the DLB format by using file management software (OL FM software, Biolog, Haward, CA, USA). The DLB format files were organized and separated into control and treated DLB format files. The average data value of the area under the kinetic curve from two independent experiments for each PM-M plate was generated by parametric software (OL PM software, Biolog, Hayward, CA, USA) and generated results between controls and treated samples were compared [35]. A median (M) value of the area under the curve was calculated for each plate in order to determine significant wells of the PM-M plate. This median value was set in the OL PM software and significant wells with a higher value of the area under the curve than the calculated median was highlighted for each plate. The median value for each plate was calculated using the following equation [35]:

(1) $$M=\frac{X+Y}{2}$$

The additional reference [35] is as below:

[35] Yu, R.M.C. Genotypic and metabolic phenotype changes in breast cells using gold-based compound cancer treatment. Doctoral thesis, Universiti Putra Malaysia, Malaysia, August 2018.

This addendum does not cause any changes to the results or conclusions in the original published paper. The authors would like to apologize for any inconvenience caused to the readers by these changes.
