# Supplementary figures and images for: Cellular Metabolic Profiling of CrFK Cells Infected with Feline Infectious Peritonitis Virus Using Phenotype Microarrays
Source: Pathogens. 2020 May 25;9(5):412. doi: 10.3390/pathogens9050412 (PMC7281222; doi:10.3390/pathogens9050412)

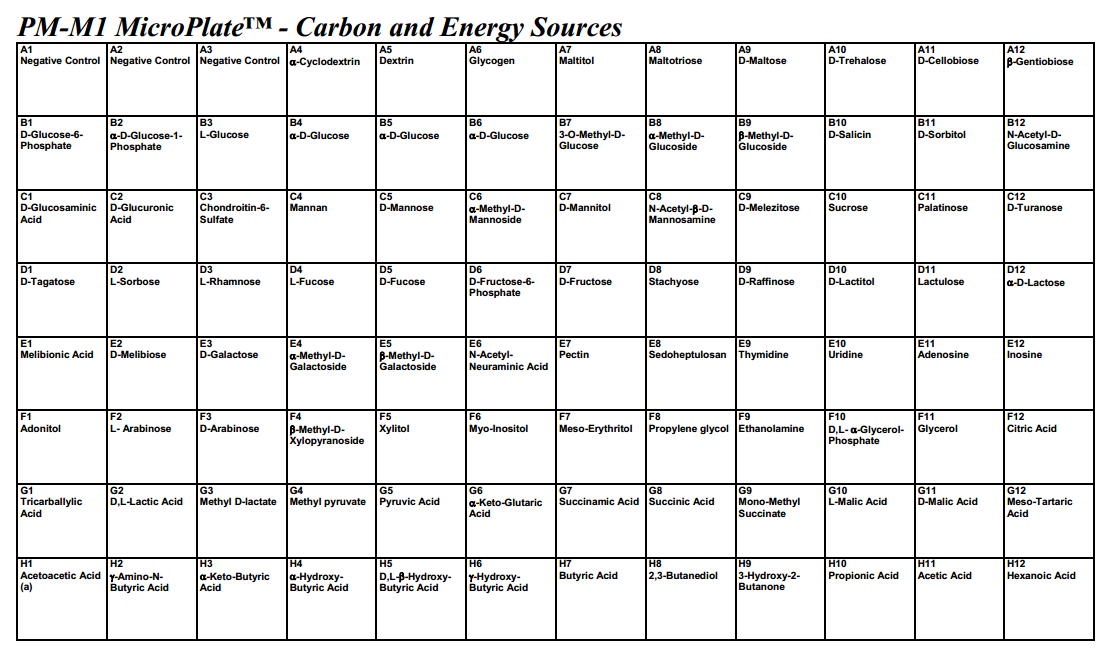

Supplement: Supplementary file 1 [file pathogens-09-00412-s001.zip › Figure S1.tif]

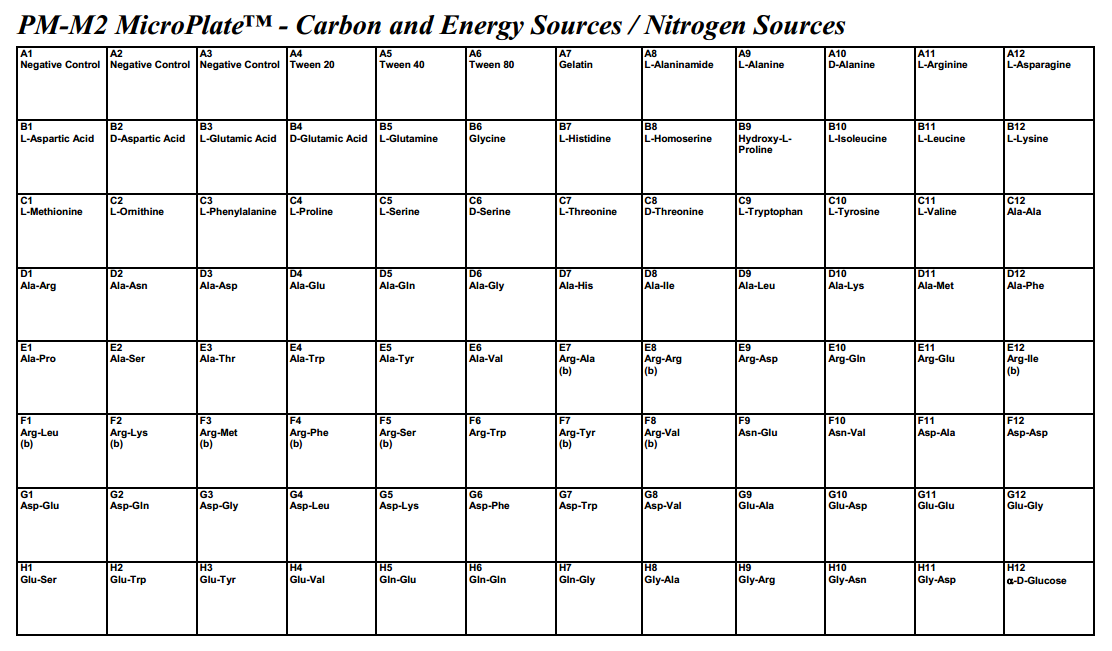

Supplement: Supplementary file 1 [file pathogens-09-00412-s001.zip › Figure S2.tif]

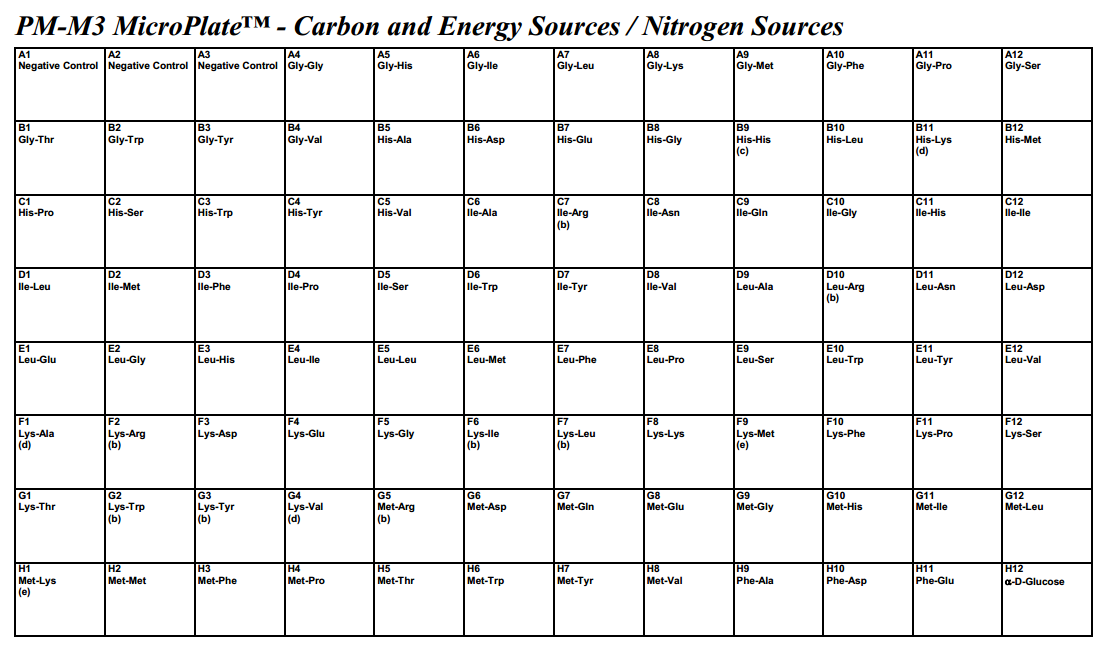

Supplement: Supplementary file 1 [file pathogens-09-00412-s001.zip › Figure S3.png]

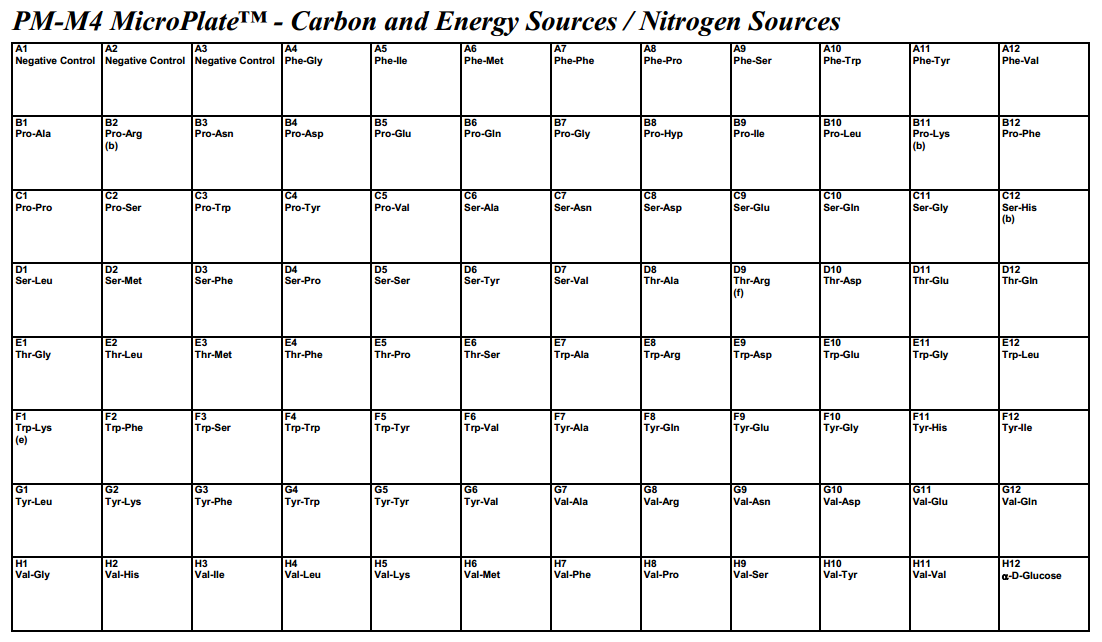

Supplement: Supplementary file 1 [file pathogens-09-00412-s001.zip › Figure S4.tif]
